# A multi-site exploration of barriers faced by vulnerable patient populations: a qualitative analysis exploring the needs of patients for targeted interventions in new models of care delivery

**DOI:** 10.1017/S1463423618000385

**Published:** 2018-07-28

**Authors:** Bobbie Johannes, Deanna Graaf, Barbara Blatt, Daniel George, Jed D. Gonzalo

**Affiliations:** 1 Research Assistant, Department of Health Policy and Administration, Pennsylvania State University, University Park, PA, USA; 2 Patient Navigation Coordinator, Office of Medical Education, Penn State College of Medicine, Hershey, PA, USA; 3 Systems Navigation Curriculum Manager, Office of Medical Education, Penn State College of Medicine, Hershey, PA, USA; 4 Associate Professor, Department of Humanities, Penn State College of Medicine, Hershey, PA, USA; 5 Associate Dean for Health Systems Education, Penn State College of Medicine, Hershey, PA, USA

**Keywords:** primary care, vulnerable patients, healthcare delivery, patient barriers, population health

## Abstract

**Aim:**

To investigate which populations of patients are considered ‘vulnerable’ across varying clinical sites, and to identify the barriers encountered by these patient populations limiting optimal health.

**Background:**

Vulnerable patient populations encounter diverse barriers that limit their ability to successfully navigate the health system, potentially resulting in poor health outcomes. Little current-day work has described types of barriers encountered by vulnerable patient populations across numerous clinical sites and settings, which is necessary to ensure health systems can begin to improve quality and disparities for all patient populations.

**Methods:**

An inductive content analysis was performed based on field-site notes and digitally recorded telephone interviews with providers/leadership at clinics/programs related to patient- and clinic-needs from January 2014 through May 2015. Using thematic analysis with grounded theory techniques, authors identified categories and themes. In total, 30 diverse clinical sites/programs including inpatient- and outpatient-based clinics providing medicine and surgery-based services were assessed through both site visits and follow-up telephone interviews. Follow-up interviews were conducted with one individual in various positions within sites/programs, including physicians (*n*=15), registered nurses (*n*=8), clinic managers/coordinators (*n*=2), clinical program coordinator (*n*=1), and care coordinator (*n*=1); one participant represented three clinical sites.

**Findings:**

In total, 30 sites/programs (*n*=30) received both a site visit and follow-up interview. Commonly reported vulnerable patient populations included those with multiple chronic conditions, lower socioeconomic status, patients in a specific stage in the continuum of care, and patients with over- and under-utilization of resources without a clear etiology. Themes related to barriers included systems barriers (eg, insufficiencies of care processes), clinic barriers (eg, lack of resources), patient-related barriers (eg, housing, transportation), and provider-related barriers (eg, inadequate time and knowledge).

**Conclusions:**

These results provide a framework to identify systems- and clinic-related barriers that can be used in population health management strategies aimed at improving health disparities within clinically diverse sites.

## Background

Healthcare in the United States is often complex, uncoordinated, and inefficient, resulting in high costs and disparities for vulnerable patient populations (Berwick *et al*., 2008; Berwick and Hackbarth, 2012). The Institute of Medicine, along with several accrediting organizations, have recommended that healthcare reform focus on the provision of optimal care for everyone by having institutions address patients’ needs in their homes, communities, and along the continuum of care, rather than through clinical care alone (Institute of Medicine (US) Committee on Quality of Health Care in America., 2001; Institute for Healthcare, 2009; Brenner, 2013a; Brenner, 2018). Influenced by policy initiatives, including the Affordable Care Act, new models of care delivery (eg, patient centered medical homes and accountable care organizations) are shifting focus from care for individual patients to managing patient populations cared for by health systems (Berwick, 2011; Kongstvedt, 2013; Haas, 2018). These new initiatives seek to improve quality by addressing population needs spanning numerous diseases and geographic areas (Berwick, 2011; Aysola *et al*., 2013; Simonetti *et al*., 2014; Adepoju *et al*., 2015; Fund, 2015).

A requirement in the design and implementation of new initiatives to improve quality is a clear understanding of the barriers encountered by patients. Previous literature has identified the barriers encountered by patient groups when attempting to access the healthcare system. For instance, patients with newly diagnosed diabetes mellitus encounter issues with healthcare coverage; Medicaid enrollees face barriers related to timely access to primary care compared to the privately insured; female veterans have been found to delay or forego care due to lack of affordable care, work obligations, caregiver responsibilities, and transportation challenges; and rural patients often face unique challenges related to provider access and transportation (Burge *et al*., 2000; Brems *et al*., 2006; Washington *et al*., 2011; Cheung *et al*., 2012). Additionally, patients at discrete points in the continuum of care encounter-specific barriers. Patients recently discharged from the hospital commonly experience challenges related to cost, insurance, transportation, and accessibility of a primary care provider (Hardman and Newcomb, 2016). Elderly mental health patients face racial and ethnic differences when seeking treatment, including issues of cost, discomfort speaking with a professional, confidentiality concerns, and difficulty obtaining an appointment (Sorkin *et al*., 2016). Lastly, many patient-encountered barriers, such as health insurance coverage, provider access, transportation issues, language barriers, and ethnic background, have been associated with lower self-rated health scores (Hong *et al*., 2004). Moreover, interventions such as patient navigation are an important factor for overcoming barriers related to cancer treatment, especially breast cancer (Elrafei *et al*., 2013; Stanley *et al*., 2013; Katz *et al*., 2014; Ramachandran *et al*., 2015). Delays in screening and follow-up care often lead to later diagnosis of cancer – patient navigators help patients overcome these barriers to ensure timely care (Stanley *et al*., 2013).

Collectively, these studies highlight barriers experienced by individual patient groups, in particular from care settings outside of the United States. However, relatively less contemporary work has explored a more comprehensive assessment of barriers encountered by vulnerable patient populations from across diverse clinical sites (ie, the perspective from which health systems will increasingly use to improve population health). Although generally accepted that high-risk patient populations encounter diverse barriers that limit their ability to successfully navigate the health system, we identified only several studies that explored barriers across patient populations in a way that enables system-level intervention. Brenner (2013b) identified that social and systems barriers, such as limited behavioral health resources or housing issues, can potentially lead to delayed diagnosis and treatment, resulting in poor health. Hirmas Adauy *et al*. and Powell *et al*. evaluated barriers across patient populations and at the system level. However, Hirmas Adauy *et al*. (2013) conducted a literature review using results primarily derived from developing countries, and clinic type was not identified. The study by Powell *et al*. (2016) conducted focus groups with interprofessional team members within Philadelphia health systems. However, inner-city residents may face different barriers to care than patients in other socioeconomic or geographic areas. Patients outside of major cities may interact with many different facets and sectors of varying health systems. These social determinants of health and patients’ healthcare utilization should be considered simultaneously to ensure health systems can begin improving quality and reducing disparities for all patient populations (Kilbourne *et al*., 2006; Marmot *et al*., 2008; Freeman, 2012; Powell *et al*., 2016).

In this study, we used field notes from clinical site visits and follow-up key informant interviews (*n*=30) with providers and leadership from a diverse range of clinical sites and programs to identify: (1) patient populations considered vulnerable across clinical sites, and, (2) barriers to care encountered by these patient populations. We believed these results would assist in the development of an intervention for vulnerable patient populations and barriers encountered by patients to be used in population health management strategies in diverse settings.

## Methods

### Study design and approach

To advance our understanding of barriers encountered by the vulnerable patient populations at diverse clinic sites, we used an inductive qualitative analysis of data obtained from 30 clinical site visits and interviews. Data collection was triangulated from two primary sources: (1) written reflections completed immediately following site visits to clinics, and (2) key-informant telephone interviews with clinical site leaders. Triangulation is a method used in qualitative research to check and establish validity by analyzing a research question from multiple perspectives (Patton, 2002; Creswell, 2009). Relevant literature on clinically based implementation research was used to inform the survey guide and probing questions (Appendices 1 and 2). We expanded upon Kilbourne *et al*.’s definition of vulnerable patient populations to include individuals who have historically experienced ongoing bias and discrimination, transient populations, and potentially mutable factors of vulnerability, including income and social networks (ie, social determinants of health) (Kilbourne *et al*., 2006; Marmot *et al*., 2008; Powell *et al*., 2016). For the purposes of this study, we defined a ‘barrier’ as anything inhibiting optimal patient outcomes. Although several studies highlight specific types of patient barriers to care, these findings tend to focus on specific disease processes and relate to care delivery outside of the United States. Therefore, we opted not to perform a formal literature review, and chose our methods to provide an assessment of barriers in this diverse sampling of sites. For these reasons, we used a data-driven inductive approach in the questions and interviews; however, we were sensitized to the healthcare disparities research framework proposed by Kilbourne *et al*. and the social determinants of health (Kilbourne *et al*., 2006; Marmot *et al*., 2008). Knowing that barriers to care exist, interviews were chosen rather than surveys to explore the research questions in detail. The Penn State Hershey Institutional Review Board approved this study as exempt research (STUDY00000027).

### Study setting

In 2013, a major curricular redesign, including the implementation of a Systems Navigation Curriculum, was initiated at Penn State College of Medicine (Hershey, PA, USA) (American Medical Association, 2013; Gonzalo *et al*., 2017a). Working in unison with a Science of Health Systems Course for first- and second-year medical students, the curriculum included an explicit experiential role for students to add value to patient care by serving as patient navigators within health systems in south-central Pennsylvania (Gonzalo *et al*., 2016a; 2016b; 2017b). A health system refers to the provision of healthcare within hospital networks and/or their affiliated hospitals or outpatient clinics, in addition to stand-alone clinics without any affiliation to a larger network. Patient navigation (PN), founded in the 1990s, is a barrier-focused, patient-centered intervention that uses outreach workers to explore barriers to care and help facilitate patients through complex, uncoordinated, and inefficient processes that result in vulnerabilities and disparities (Freeman, 2012; Brenner, 2013a; Balderson, 2014). The research team embarked on identifying and developing a network of clinical sites where students would be able to fulfill these roles (Gonzalo *et al*., 2017a). The needs assessment (ie, vulnerable patient populations and their barriers to care) occurring during the development of the PN network provided the opportunity to answer the research questions explored in this study.

### Clinical site sampling

We performed a diverse sampling of clinical sites to identify vulnerable patient populations and identify interventions that could be implemented in various clinical sites. We identified sites through known relationships with our leadership teams, and an advisory board of community and health systems leaders. The reasons for site referrals for potential inclusion were varied, and included perceived challenges experienced by patients receiving care within the clinic and the possible opportunity for additional assistance of the health system to patients. We sampled as many sites as allowed by resource limitations, with a main goal to identify enough sites for student placements. The sampling number was not selected based upon data saturation, and we made an *a priori* decision to analyze all collected data regardless of when saturation was reached. Sites were contacted via email or telephone, requesting a site visit with their site leadership team. Besides distance from the College of Medicine (and primary hospital, Penn State Milton S. Hershey Medical Center), we did not exclude clinical sites from investigation based on any pre-determined factors. The diversity of sites would allow us to identify similar barriers that each site and/or program experiences in order to implement an appropriate intervention.

### Data collection

To ensure the credibility of our results, we triangulated data from two sources and across several clinical site types: clinical site visits by members of the research team and 1:1 telephone interviews with site leadership (Shenton, 2004). From January 2014 to May 2015, at least two members of the research team conducted site visits to discuss services provided, patient population demographics, barriers encountered, and site needs (Appendix 1). Following each site visit, two team members typed a 1–2 page reflection about the visit, specifically addressing patient populations served and site needs. Next, within two weeks of each visit, a member of the research team performed a semi-structured, audio-recorded telephone interview with at least one site leader, including physicians, clinic managers, patient navigators, care coordinators, or medical directors. The interview guide included both closed- and open-ended questions related to vulnerable patient populations, with specific probes related to PN presence (Appendix 2). Interviews were transcribed verbatim by a professional transcriptionist.

### Data analysis

Data were analyzed using thematic analysis incorporating several widely used grounded theory techniques (Boyatzis, 1998; Braun and Clarke 2006). During data collection, investigators took notes, and, using the process of constant comparative analyses, identified initial categories and themes. A preliminary codebook was created to facilitate subsequent analysis. The research team collaboratively analyzed three transcripts to mutually agree upon initial codes and modify the codebook. Then, two investigators (B.J. and D.G.) independently coded several transcripts, comparing codes for inconsistency and agreement, followed by regular joint adjudication sessions and updating of the codebook. Remaining transcripts were then independently coded with regular adjudication sessions. Disagreements were discussed and codes were created and collapsed, resulting in an iteratively updated codebook. Researchers used Computer Assisted Qualitative Data Analysis Software, NVivo 10 QSR International, to manage, outline, and understand unstructured information. The research team discussed and came to consensus on overarching categories, themes, and exemplary quotations. As identified below, our results showed resonance with the existing literature related to barriers to care, suggesting these results are transferrable to other clinical settings (Shenton, 2004). We developed a conceptual framework characterizing the relationship between barriers, vulnerable patient populations, outcomes, and potential interventions.

## Results

### Clinical site demographics and participant characteristics

During the study period, 30 clinical sites and programs were assessed through both site visits and follow-up telephone interviews. Characteristics and a description of each site are shown in Table 1. Sites were highly variable, including both inpatient- and outpatient-based clinics providing medicine and surgery-based services. Site visits involved a 1–2 h meeting (range 1–7 h) with several individuals from the clinic/program or health system. Follow-up interviews were conducted with one individual in various positions within sites/programs, including physicians (*n*=15), registered nurses (*n*=8), clinic managers/coordinators (*n*=2), clinical program coordinator (*n*=1), and care coordinator (*n*=1); one participant represented three clinical sites. Below is a description of the themes and representative quotations.

**Table 1:** Characteristics of clinical sites and programs assessed for potential medical student systems roles (*n*=30, 2013–2015)

		No. sites by affiliation		
Programs/sites	Brief description of programs and/or clinical services	University	Community	Independent
Inpatient setting				
Acute Rehabilitation Hospital Transitions Program	Facilitates the transition for patients requiring acute rehabilitation for spinal cord and brain injury to other care settings	1		
Emergency Department	Triages, admits, and/or discharges patients with the full spectrum of medical issues for pediatric and adult patient populations, requiring general and specialty care	1		
Internal Medicine Service Discharge Program	Integrated, team-based transitions program that engages and facilitates patients’ care issues during the transition from hospital-based medicine units	1	2	
Psychiatric Hospital Discharge Program	A five-week follow-up program that assesses patients post discharge to ensure adherence to care plans with the goal of decreasing readmissions and improving outcomes	1		
Rehabilitation Hospital Transitions Program	Guides spinal cord and brain injury patients through the recovery process by setting goals and adjusting their lifestyle to produce the best outcomes			1
Skilled Nursing Transitions Program	Facilitates the safe, timely, and efficient transition of care from the skilled nursing center or less intensive rehabilitation setting to other facilities or home	1		
Surgical-Oncology Transitions Program	Manages same-day or brief inpatient surgical procedure patients along the continuum to ensure appropriate coordination of care activities upon discharge	1		
Outpatient setting				
Breast Cancer Program	Provides medical, social, and behavioral services to breast cancer patients following the definitive treatment phase of malignancy, or the ‘survivorship phase,’ of their illness	1		
Clinic for Stable Workers	Provides primary care services to underinsured stable workers employed by a local horse racetrack			1
Clinic for Underserved Patients	Provides physical assessments, education, and other healthcare services (eg, dental services) to underserved and uninsured patients			3
Insurance Provider	National, diversified health care partner that serves the regional community by offering health and dental insurance, and vision care; identifies at-risk and superutilizer patient populations to improve outcomes			1
Heart Failure Clinic	Assists in managing patients with their illness through regularly scheduled visits and navigating care plans		1	
HIV Clinic	Provides diagnostic, therapeutic, and supportive services related to HIV, including education about disease and transmission, treatment recommendations, and access to care		1	
Inflammatory Bowel Disease (IBD) Clinic	Specialty service with a collaborative care model accepting patient referrals to diagnose and treat patients with IBD	1		
Internal Medicine Clinic	Multi-disciplinary team that provides primary and specialty care and manages prevention, diagnosis and treatment of chronic conditions	1		
Family Practice Outreach Program	Primary care program that aids high risk patients through continuum of care through follow-up phone calls, home visits, and care coordination			1
High-Risk Outreach Clinic	Interdisciplinary care team that coordinates community care for high-risk patients by working to resolve patient barriers including transportation, insurance, and social issues		1	
Patient-Centered Medical Home	Primary care that emphasizes care coordination and communication while providing medical care to pediatric and adult patient populations	4	1	
Spine Clinic	Joint surgery and spinal program working in a multi-disciplinary team approach that provides a comprehensive assessment and treatment plan for patients	1		
Surgical Weight Loss Program	Multi-disciplinary pre-surgical program that provides education and evaluation needed to prepare patients for surgical weight loss surgery	1		
Tuberculosis Clinic	State-run clinic that provides care to patients with acute and chronic tuberculosis, specifically by addressing patient needs both in clinic and during home visits to ensure adherence to care plans			1
Palliative Care Program	Integrated with the Cancer Institute, the program provides specialized medical care to patients with serious illnesses, seeking to improve quality of life for both the patient and the family	1		
Total		16	6	8

### Vulnerable patient populations

Among the varying clinic types, the analysis identified several vulnerable patient populations in need of additional services (Table 2). Most frequently identified patient populations included:

1. Patients with multiple chronic conditions, such as diabetes mellitus and congestive heart failure;
2. Patients of lower socioeconomic status or neighborhood that may be underinsured, homeless, and/or financially unstable; and
3. Patients who over- or under-utilize healthcare resources, for unclear reasons.

**Table 2:** Clinic leadership perceptions of vulnerable patient populations in their clinical sites (*n*=290)

Category	Representative examples	Frequency of code references^a^ [290 (%)]
Multiple chronic conditions	Patients with chronic conditions (eg, diabetes, CHF, CVA, autoimmune diseases) Patients with conditions requiring multiple changes in therapy and/or specialists Patients (eg, children) who are developmentally delayed and need regular follow-up Patients with cancer lesions requiring longer-term follow-up Patients with complicated medical history and unstable conditions	84 (29%)
Low socioeconomic status or neighborhood	Patients who are on medical assistance, uninsured, or underinsured Patients who are homeless, or living in poor housing (eg, horse stable workers) Patients who are ‘afraid’ to take off work due to fear of job loss Patients who live in high-risk crime areas Patients who are considered ‘transient,’ limiting establishment of care Patient with poor health literacy	52 (18%)
Specific stage in continuum of care or age group	Patients who are in the preoperative disease stage (eg, surgical weight loss) Patients who are in the postoperative disease stage (eg, breast cancer surgery) Patients who are elderly or ‘geriatrics’ patients Patients transitioning from one care setting to another (eg, hospital to outpatient)	36 (12%)
Over- and under-utilization of health care services without clear reason	Patients who have high utilization of services (eg, readmissions, ED visits) Patients who are non-adherent with care plans/medications Patients with a high drop-out and no-show rate in clinic Patients with challenging and complex social situations Patients with ‘high intensity’ needs within clinic	32 (11%)
Mental health issues	Patients with psychological issues (eg, anxiety, depression, bipolar, schizophrenia) Patients with behavioral or emotional issues Patients with substance abuse disorders	29 (10%)
Cultural/ethnic groups	Patients who are considered undocumented individuals or refugees Patients who are primarily non-English speaking Patients who are members of Hispanic populations	28 (10%)
Inadequate social support/environment	Patients who are alone and overwhelmed with their disease Patients without family support Patients who were previously or recently incarcerated	11 (4%)
Level of emotional readiness to accept care	Patients who are prideful Patients who are overly anxious about their disease	10 (3%)
Low access	Patients who have poor access to community resources (eg, nutrition services) Patients who are new to ‘free clinics’ who have not received sufficient care Patients without a primary care provider Patients with difficulty arranging appointments with specialists (eg, psychiatrist)	8 (3%)

a Code references indicate the number of times the specific code was identified or ‘referenced’ in the analysis. For example, if ‘multiple chronic conditions’ was identified in detail, the code may have been reference more than once. CHF=congestive heart failure; CVA=cerebrovascular accident; ED=emergency department.

Specific patients who either over- or under-utilized healthcare services (eg, several hospital readmissions) were identified as a group with an underlying issue(s) or challenge, making these patients vulnerable to poor outcomes. However, participants often could not explicitly identify reasons for the observed utilization issues.

Other populations included patients in a specific stage in the care continuum, patients with mental health issues or with a particular level of emotional readiness to accept care, members of particular cultural and ethnic groups, patients with inadequate social or environmental support, and patients with poor access to the healthcare system.

*‘Based on the population we have [in our clinic], patients with a complicated medical history, [with] unstable conditions that are under managed or not well managed [are vulnerable].’*

*‘They come here because they’re homeless. Often they have no medications or haven’t had any insurance for a while to get medical care.’*

*‘The majority of our geriatric patients [are vulnerable]. Patients that have multiple complex medical conditions who often times have limited health literacy. Often times they’re on multiple medications, [which] pose a lot of questions for the patient, and concerns for adverse side effects.’*

### Barriers to care

The analyses identified four key categories related to barriers that limit ideal care: (1) systems barriers, (2) clinic barriers, (3) patient barriers, and (4) provider barriers (see Tables 3 and 4).

**Table 3:** Clinic site leadership perceptions of systems barriers limiting patient outcomes in their clinical sites (*n*=620)

Category	Code references [*n* (52% of 620)]	Representative barriers
Insufficiencies in system	109 (17%)	Patients discharged without follow-up appointment arranged prior to discharge (‘patients fall through the cracks’) Patients have long wait times for a referral appointment to a specialist or primary care physician Lack or insufficient community resources Health information exchange via electronic health record does not allow sharing of information between sites Health insurance challenges that make eligibility process difficult (eg, six months to complete bariatric weight loss program) Multiple provider and specialist provider appointments occur at different times and in different locations, resulting in increased patient burden Transition from hospitalized to outpatient status are difficult due to insufficient resources HIPPA and patient privacy tends to inhibit the care process due to the need to have an agreement signed in order to discuss a patient
Provider or service access	103 (17%)	Lack or absence of sufficient service access (eg, mental health, education, emotional support/counseling, home health services) Lack or absence of primary care clinic access in the area or region Lack or absence of consultant services in area/region Insufficient services due to the increasing age of the US population Lack of sufficient provider time (eg, providers only have a maximum of 20 min with patients)
Insurance access	55 (9%)	Medical assistance is difficult to obtain without provider and/or legal help Patients are often times ineligible for state or federal insurance plans even though they are without another form of insurance Patients who have some type of insurance are often underinsured and still cannot afford healthcare services and/or medications Having multiple payers or multiple insurers causes confusion for patients in terms of where and how they can access healthcare services
Medication issues	38 (6%)	Medication reconciliation creates challenges between multiple providers caring for a single patient Numerous medications prescribed for multiple conditions (eg, polypharmacy) creates a greater risk for harm to patients Issues in authorization of medications creates an added patient burden Medications are often too costly for patients to afford, and there is not adequate funding for them to purchase these medications
Communication breakdowns	17 (3%)	Poor communication between providers (eg, within and between clinics) Poor communication between providers and patients (eg, rehab facilities, inpatient discharge, etc.)

HIPPA=Health Insurance Portability and Accountability Act of 1996.

**Table 4:** Clinic site leadership perceptions of clinic, patient, and provider-related barriers limiting patient outcomes in their clinical sites (*n*=620)

Category	Code references [*n* (48% of 620)]	Representative barriers
Clinic barriers	143 (23%)	Long wait times for and difficulties in arranging follow-up appointments in clinic Insufficient staff numbers (eg, appointment times are too short, providers unavailable) Clinic hours are irregular and do not suit patient needs Inconsistencies in work flow (eg, inconsistent documentation, break downs in patient charting) Technology not up-to-date (eg, paper charting) Limited resources within clinic (eg, teaching tools) Limited capacity for patients in terms of space within clinic Patients are required to fulfill staff roles (eg, requesting documentation from other providers) Clinic rooms are not functional as exam rooms (eg, no sink in room) Geographic locations of clinics within the same provider network are a great distance from each other
Patient-related barriers	138 (22%)	Perceived non-compliance with recommendations and plans Lack of adequate housing Poor finances (eg, inability to pay electric bills, out of work, cannot make mortgage payments, unable to afford healthy foods) No transportation to attend clinic appointment Lack of health literacy (eg, understanding clinical processes), patient speaks different language than clinic providers Co-morbidities make it difficult to follow care plans Patients are often ‘down on their luck’ The stigma associated with mental health, and addiction creates a barrier to care Patients often become overwhelmed with their care plan for financial reasons or others
Provider-related barriers	17 (3%)	Providers have limited time to address patients’ needs Providers are unaware of patients’ social issues at home Providers are unaware of resources that are available to help their patients Providers have a lack of understanding of different cultural backgrounds Providers practice patient-directed decision making rather than patient-centered care Patients feel that they cannot trust their physician Providers are unaware of prescription costs, and patients’ inability to afford medication s/he has prescribed

#### Systems barriers

We defined a systems barrier as any factor related to resources or services within the healthcare setting, the accessibility to healthcare services and current governmental policy (eg, insurance eligibility) that negatively influences or affects his/her ability to access healthcare and/or benefit fully from services. Systems barriers accounted for the majority of identified barriers (*n*=321, 52% of total; Table 3), and were classified as *insufficiencies within the health* (*n*=109); *provider or service access* (*n*=103); *insurance access* (*n*=55); *medication issues* (*n*=38); or *communication breakdowns* (*n*=17).

*‘Within the silos, our patients have needs in mental health, housing, finances, transportation, access to understanding medications and medical literacy. There are limited support services within the community, here in [south-central PA] and nation-wide.’* (Insufficiency within health system)

*‘Getting a psychiatric or mental health appointment can be a real challenge. There are not enough mental health providers. Sometimes they can get in for a therapist appointment sooner but to see a psychiatrist often it is two months.’* (Provider or service access)

*‘[Patients] run out of insurance for one reason or another, because they didn’t renew it, because they changed places where they were living and the paperwork never reached them.’* (Insurance access)

*‘A big systems barrier is medication; understanding what the [medications] are that [patients are] on, and the importance of taking them, [as well as] being able to afford their own medications.’* (Medication issues)

*‘There can be communication challenges. Communication channels between all of these providers are multi-fold, usually involving chart messaging, emails, and periodic team meetings, held mainly at [local clinic].’* (Communication breakdowns)

#### Clinic barriers

We defined a clinic barrier as any factor within a clinic that delays a patient’s ability to receive healthcare and/or benefit fully from services. Clinic barriers were the second most frequently identified barrier to ideal patient outcomes (*n*=143, 23% of total; Table 4). Leadership identified factors related to care delivery processes or the structure of clinics that can potentially have a negative impact on patient outcomes. Participants commented on long wait times and difficulties arranging follow-up appointments, insufficient staff, irregular clinic hours that are not conducive to patients’ schedules, out-of-date technology (eg, paper charting), inconsistencies in work flow, and limited resources within the clinic rooms.

*‘Six months of therapy is recommended, and if a patient doesn’t call back, we tend to not have adequate follow-up to call them—so that’s a potential loss.’*

#### Patient-related barriers

We defined a patient-related barrier as any factor from one’s social environment, such as interpersonal and individual self, that negatively affects or influences a patient’s ability to access healthcare and/or benefit fully from services. These patient-related barriers are often identified as social determinants of health (Marmot *et al*., 2008). Participants identified several factors related to patient’s lives or situations that limit ideal outcomes (*n*=138, 22% of total; Table 4). Several respondents commented on perceived non-adherence with care plans, lack of adequate housing, poor finances, language barriers, transportation issues, low health literacy, the stigma of mental health, and addiction issues.

*‘There are sometimes other challenges [patients] have besides the medical arena. They may have problems with supporting housing, with keeping the heat or the electricity on in their house, they may not have work, some of our patients have been evicted and their basic needs aren’t being met.’*

*‘[One patient] came down with a knee problem and it turned out to be a severely degenerative joint that needs to be replaced and she just feels that she can’t take off work because then she won’t have any money to pay her mortgage.’*

#### Provider-related barriers

We defined a provider-related barrier as any factor related to individual or groups of providers, such as knowledge, interpersonal skills, culture, or clinical circumstances with the potential to negatively influence a patient from fully benefiting from services. Participants identified several provider-related factors that may limit ideal patient outcomes (*n*=17, 3% of total; Table 4), including insufficient time to address patients’ needs, lack of awareness about patients’ social situations and available resources, lack of skills in culturally competent care, patient-directed (eg, autonomous provider) decision-making rather than patient-centered care, and trust issues.

*‘I think some of them are cultural issues…different clinicians may need a little sensitivity.’*

## Discussion

In this study, providers and leadership in clinical sites and programs in several diverse health systems and clinics identified vulnerable patient populations in need of additional medical care, including patients with multiple chronic conditions, lower socioeconomic status, and patients who disproportionately (under- and over-) utilize resources. Notably, patient groups were not primarily identified by a specific disease (eg, patients with congestive heart failure) but rather included groups with multiple or non-disease-specific issues (eg, patients with congestive heart failure, diabetes mellitus, with poor financial support and living in a low socioeconomic neighborhood). Numerous types of barriers were also identified, many of which were independent of patient circumstances, and related to the system and clinic context, highlighting areas for potential systems improvement. Despite site heterogeneity, perceived system and patient-related barriers were similar across sites. These results inform a more comprehensive conceptual framework of vulnerable patient populations, and potential barriers they encounter from various clinical settings across several health systems.

**Figure 1:**
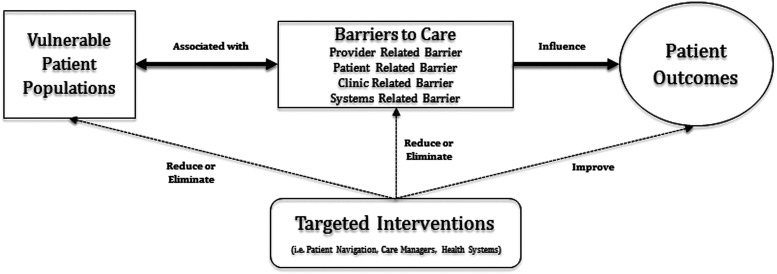
Conceptual framework of barriers to care and vulnerable patient populations. It depicts how barriers to care, identified in this study, create vulnerable patient populations across the spectrum of care, which influences patient outcomes. Targeted interventions can potentially have a mediating effect on barriers to care, vulnerable patient populations, and patient outcomes by reducing, eliminating, or improving these variables.

This analysis is consistent with the theoretical framework initially developed by Kilbourne *et al*. (2006) related to health disparities research, which includes the following three phases: (1) detection of vulnerable patient populations, (2) understanding why disparities exist, and (3) reducing or eliminating disparities. Our work focuses upon the first two phases, in the context of a regional patient population across a number of clinically diverse sites (Kilbourne *et al*., 2006). As proposed by Kilbourne *et al*., the underlying manifestation of vulnerable populations originates from several key determinants, including the health system (eg, organization, delivery), patients (eg, beliefs, culture), and providers (eg, attitude, competing demands). Our results validate this conceptual framework (Figure 1) as we identified similar categories, including the partition of ‘healthcare system factors’ into those that are related to particular clinical sites and factors outside of those sites. Additionally, inasmuch as our participants’ perspectives are representative of true barriers associated with disparities, our results may suggest disparities are most frequently associated with health systems factors, more so than patient or provider factors. Several studies have identified certain disparities that are attributable to ‘organizational characteristics’ of the health system, such as complexities of communication and resources (Tarlov *et al*., 1989; Greenfield *et al*., 1995). However, these studies relate directly to a specific disease (eg, diabetes mellitus), a specific unit (eg, intensive care unit), or are studied from higher-level claims data, all of which limit the ‘on-the-ground’ perspective of frontline providers and clinical site leaders as to the issues encountered in their local environments. These results advance an understanding of potentially mutable health system factors that could decrease disparities across non-disease-specific populations (ie, the focus of population health management). Future studies will need to further quantify the relative importance of each barrier type to disparities in different clinical settings.

Several vulnerable patient populations were challenging to categorize, which highlighted the nuanced and overlapping challenges health systems face in addressing the numerous factors impacting health. In particular, the category labeled ‘over- and under-utilization of health care services without clear reason’ is a group of patients identified as in-need, but with ambiguous underlying cause(s) driving more visible resource utilization (eg, hospital readmissions) or more pronounced utilization (eg, first healthcare visit in an extended period of time). This struggle reflects the intertwined relationship between patients, their challenges, and the ways in which they interact with fragmented health systems. Previous research has identified that 1% of patients account for over one-fifth of all healthcare spending, and 5% of patients – commonly referred to as ‘superutilizers’ – use nearly 50% of the total number of services and healthcare costs (Johnson *et al*., 2015). As health systems transition toward population health management, there is an increasing need to focus on ‘superutilizing’ patients to limit spending and curb costs. However, as suggested by our results, the root causes of impediments to optimal care are not always clear, even to those providers seeking to optimize their care. Tailored patient-centered investigations may be required in different patient groups to uncover the reasons for superutilization.

The current literature makes several references to barriers that impede patients’ ability to successfully access care; however, these barriers are not well defined or classified. Brenner (2013a; 2013b) alludes to approximately 50 barriers patients face but these are not further classified. Aligned with the World Health Organization’s definition for universal healthcare, a literature review by Adauy *et al*. (2013) identified acceptability, accessibility, availability, and contact as the major barriers to care internationally (World Health Organization, 2010; Hirmas Adauy *et al*., 2013). This review also identified the most frequent barriers as drug costs, medical consultations and examinations, fear or shame of receiving care, suspiciousness in providers and treatments, social stigma, beliefs and myths (Hirmas Adauy *et al*., 2013). However, this study used data primarily from clinical care settings in underserved countries. Powell *et al*. (2016) identifies patient level barriers within a US health system, however, the participants are not those who are in the position to make necessary changes to population health management (Powell *et al*., 2016). Our results identified several similar major barriers to studies done by Jackson *et al*. (2001), Powell *et al*. (2016), and Kilbourne *et al*. (2006), and also allowed for further categorization of these barriers into the categories proposed by Kilbourne. Delineating the distinction between systems, clinic, and individual (patient and provider) barriers is critical in health systems’ use of segmentation approaches to improve health outcomes and costs by identifying patients who might be able to avoid health problems with greater support (Fund, 2015).

With the increasing number of insufficiencies being identified in health systems, including disproportionately high rates of disparities, increasing cost, and preventable patient safety events, specific interventions have been proposed and piloted to improve outcomes. Over the past 20 years, PN has been evaluated in several oncology settings, demonstrating positive results, but there have been limited studies of PN in other patient settings. Pilot work using PN in hospital discharge programs and outpatient diabetes programs have shown promise, with improvements in follow-up clinic appointments and compliance (Freeman and Rodriguez, 2011). Similarly, the concept of ‘hot spotting’ has been used to identify vulnerable patient populations based on their superutilization of resources, with the end goal of targeting their barriers to care to limit utilization (Brenner, 2018). These models have been context-specific, focusing upon patients with specific diseases (eg, cancer), geographic locations with lower socioeconomic status (eg, superutilizers in an urban setting), or during points in a patient’s care continuum (eg, hospital discharge programs) (Freeman and Rodriguez, 2011; Brenner, 2013a; Accenture, 2015). However, the principles of PN and hot spotting have the potential to complement the services provided in new models of care delivery, such as Patient-Centered Medical Homes and Accountable Care Organizations. Additionally, these results can be used in the development of new roles or outreach programs in redesign initiatives. However, no matter the explicit intervention, patients with increased access to the US healthcare system will undoubtedly accrue an increased financial burden. These challenges may ultimately influence patients to not capitalize upon interventions or sacrifice other financial needs in their daily living. Further work is needed to investigate barrier types within homogenous groups of clinics (eg, outpatient surgery clinics) in different geographic regions to further identify unmet needs for patient populations. With specific attention to patient-reported barriers, health systems can prioritize those that may be most mutable from a population health management perspective, and explore the ultimate financial and health-related impact on patients in new models of care delivery.

This study has several limitations. First, the view of ‘site leadership’ did not include patients, or in a few situations, frontline providers, which may limit the perspectives of actual vulnerable patient populations and barriers in these settings. Second, although we investigated 30 clinical sites, each varied in patient populations and services provided. Given the relatively low numbers of specific site types, such as one surgical weight loss clinic, these results are not generalizable to any one specific site type. Also, the sample was limited to clinics located in south-central Pennsylvania, and therefore may not be applicable to other geographic areas with a more variable socioeconomic status or other unidentified factors.

In conclusion, all participants identified vulnerable patient populations in need of additional healthcare beyond that already being delivered within their clinic or program. Numerous barriers were identified, many of which were independent of patient circumstances, and related to the systems and clinic context. These results highlight key areas for population health management aimed at decreasing disparities. Achieving the goals of new models of care delivery in a value-based era requires policy makers, clinic directors, and providers to address the numerous barriers that prevent patients for accessing and realizing optimal outcomes.

## APPENDIX 1

**Representative site visit interview guide**

- What are your clinic/program’s current needs in improving the health of your patients?
- Please describe the most common systems-related issues or barriers your patients may encounter. What system-related barriers are frequently addressed by patient navigation/care coordination in your hospital, health system, clinic or department?
- What value do you see in having students embedded within your clinic to assist you in achieving better health outcomes for your patients?
- What barriers might you anticipate in embedding students in your clinic/program?
- Do you have a possible/potential MD champion or mentor for the patient navigation program?

## APPENDIX 2

**Key Informant follow-up site visit interview guide**

- Which patients in your clinic would benefit most from having a medical student assigned to them in the role of patient navigators?
- Please describe the most common systems-related issues or barriers your patients may encounter. What system-related barriers are frequently addressed by patient navigation/care coordination in your hospital, health system, clinic or department?
- What value, if any, do you see in having a medical student serve in the role of patient navigator (to promote the timely movement of patient through an often complex health care continuum in your hospital, health system, clinic or department?
- Does your in your hospital, health system, clinic or department presently offer patient navigation/care coordination? If *yes*:
  - How do patient navigation/care coordination work?
  - How many patient navigators are there? Tell me about their background/training?
  - At what point does a patient navigator become involved with a patient?
  - Please describe the role that they have in your hospital, health system, clinic or department.
  - Describe how the physician/nurse practitioners coordinate with patient navigators/care coordinators in your hospital, health system, clinic or department.
  - Are physicians/nurse practitioners in your hospital, health system, clinic or department generally supportive of patient navigation/care coordination?
  - At what point would a navigator be integrated into the care of your patients?
  - What measures do you use in your clinic to assess the outcome of navigated patients?
    - What additional metrics could be used?
  - What is your source of funding, if any, for patient navigators/care coordinator positions?
  - What skills/competencies of patient navigators/care coordinators do you think are useful?
  - What training would enhance patient navigation/care coordination in your hospital, health system, clinic or department?
  - Describe how your program’s effectiveness is measured.
    - Probes:
    - How is data collected?
    - How frequently do you report your programs progress?
    - To whom, do you report progress or results?
  - Describe the champions of Patient Navigation?
    - Probes:
    - Medical or radiation oncologist
    - CEO
    - Health care providers
    - Practice managers
  If *no*:
  Would physicians/nurse practitioners in your clinic generally be supportive of patient navigation/care coordination?
- Do you track:
  - Patient satisfaction? If so, how?
  - Process measures for patient encounters, such as time to diagnostic testing, referrals to specialists, initial treatments, etc.?
  - Are you in a position to share data regarding patients in the patient navigator program with the primary research team?
- What primary functions would be most beneficial for a patient navigator to fulfill within your hospital, health system, clinic or department?
- Do you have a possible/potential MD champion or mentor for the patient navigation program?
  - Yes
  - No
- Do you have any advice or comments for us as we develop our program of systems-based practice for medical students?
